# Altered resting-state prefrontal activity and network topology in adolescents with depression: an fNIRS study

**DOI:** 10.3389/fpsyt.2026.1696556

**Published:** 2026-02-23

**Authors:** Gui Gui, Chang Shu, Xiaofen Zong, Hao Liu, Huiling Wang, Huawei Tan, Yu Bai, Maolin Hu, Min Yang, Gaohua Wang

**Affiliations:** 1 Department of Psychiatry, Renmin Hospital of Wuhan University, Wuhan, Hubei, China; 2 Hubei Provincial Clinical Research Center for Psychiatry, Wuhan, Hubei, China

**Keywords:** adolescents, depression, fNIRS (functional near infrared spectroscopy), network topology, resting-state

## Abstract

**Background:**

Adolescent depression is associated by substantial cognitive impairment and poor treatment response, yet its neurobiological underpinnings remain insufficiently understood. Resting-state functional near-infrared spectroscopy (fNIRS) offers a portable and developmentally sensitive tool to examine intrinsic prefrontal activity and network properties.

**Methods:**

Seventy-nine adolescents with depressive disorder (DD) and age-matched healthy controls (HC) underwent 6-min resting-state fNIRS recording over the prefrontal cortex. We extracted fractional amplitude of low-frequency fluctuations (fALFF) and resting-state functional connectivity were computed, and graph-theoretical metrics, including clustering coefficient, local/global efficiency, path length, and small-worldness were derived. Depressive and anxiety symptoms was assessed using the 17 - item Hamilton Depression Scale (HAMD-17) and Hamilton Anxiety Scale (HAMA), and cognitive performance was assessed with the Brief Assessment of Cognition in Schizophrenia (BACS).

**Results:**

Compared with HC, the DD group exhibited elevated prefrontal fALFF in multiple channels (e.g., ch11, ch26, ch31), reduced average functional connectivity within and between bilateral frontal regions (including FPA and Broca’s area), and lower clustering coefficient, local efficiency, and global efficiency relative to healthy controls, whereas path length and small-worldness were preserved. Region-specific associations with cognition were observed: fALFF in ch11 positively correlated with verbal fluency, whereas fALFF in ch31 negatively correlated with executive functioning, these associations remained significant after controlling for depressive and anxiety symptom severity. *Conclusions:* Adolescents with depression show elevated prefrontal fALFF and reduced network efficiency, with region-specific associations with cognitive performance. These findings suggest that resting-state fNIRS is a developmentally suitable method for probing prefrontal neurocognitive alterations in youth depression.

## 1 Introduction

Adolescent depression is a growing global health concern, with accumulating evidence indicating both high prevalence and a rising disease burden. A meta-analysis reported that approximately 21.3% (95% CI: 16.7–26.7%) of children and adolescents worldwide experience depression or elevated depressive symptoms, with moderate-to-severe cases affecting 18.9% and major depression 3.7% (1, 2). Global Burden of Disease data further reveal that among individuals younger than 30 years, the incidence and disability-adjusted life years (DALYs) attributable to depression increased by more than 50% between 1990 and 2021 (1). Consistently, longitudinal analyses document steep upward trends in major depressive disorder (MDD) burden among adolescents and young adults aged 10–24 years, underscoring the urgency of prevention and intervention (3).

Beyond mood symptoms, adolescent depression is associated with a substantial cognitive burden and long-term functional consequences, with cognitive dysfunction contributing to functional impairment and an elevated risk of relapse (4, 5). These findings highlight the need to clarify the neurobiological substrates underlying adolescent depression and their links to cognition. Adolescence represents a sensitive developmental window characterized by pronounced maturation of large-scale brain networks. Converging evidence from structural and functional neuroimaging studies indicates a developmental shift from predominantly local connectivity toward more distributed network organization, accompanied by greater modular segregation and improved communication efficiency—changes thought to support emerging executive abilities (6–8). In large youth cohorts, modular segregation increases across ages 8–22 years and mediates improvements in executive performance; structure–function coupling similarly strengthens with age along cortical hierarchies of functional specialization (7, 8).

Resting-state fMRI and graph-theory studies in MDD have repeatedly revealed large-scale network dysfunction, especially in fronto-parietal and default-mode systems, often characterized by reduced network efficiency, hypoconnectivity, or disrupted modular architecture (9–11). At the regional level, the fractional amplitude of low-frequency fluctuations (fALFF) quantifies spontaneous neural activity; alterations in fALFF have been reported in MDD and linked to cognitive deficits (12, 13). Moreover, individual differences in global efficiency—a graph metric of integrative communication—relate to intellectual performance (14, 15); complementary work shows that lateral prefrontal global connectivity predicts performance under high cognitive-control demands (16), linking network topology to behavior.

Functional near-infrared spectroscopy (fNIRS) provides a developmentally appropriate, portable, and movement-tolerant method for probing prefrontal physiology in pediatric and adolescent cohorts, particularly where MRI is less feasible. Its portability and tolerance to naturalistic settings make it particularly valuable for studying adolescent depression, including in community or school contexts (17, 18). While resting-state fNIRS remains comparatively underexplored, existing work in affective disorders has reported reduced prefrontal connectivity and disrupted network organization at rest (19). Task-based fNIRS in adolescent depression further demonstrate attenuated prefrontal activation and decreased task-related connectivity during verbal fluency, supporting fronto-cortical dysfunction in youth (20, 21). However, few studies have directly examined whether resting-state fNIRS metrics are related to standardized measures of cognition. This represents a critical gap, as cognitive dysfunction is a core feature of adolescent depression. Although several resting-state fMRI studies have linked network connectivity or low-frequency fluctuations to cognitive performance in this population (22, 23), comparable evidence from fNIRS remains limited.

The present study aims to address these gaps. Specifically, we used resting-state fNIRS to jointly examine regional spontaneous activity (fALFF) and network topology (clustering, local/global efficiency, path length, and small-worldness) in adolescents with depression compared with healthy controls. Crucially, we examined whether these neural indices are associated with not only depressive and anxiety symptoms but also with objective cognitive performance measured with the Brief Assessment of Cognition (BACS)—a standardized battery with robust psychometrics and normative data (24). By integrating neural and behavioral measures, we aimed to test whether adolescent depression is characterized by multiscale disruption of prefrontal dynamics and whether these disruptions represent behaviorally relevant markers of cognitive and affective dysfunction.

## 2 Methods

### 2.1 Participants

Adolescent patients with depression were recruited from both outpatient and inpatient services of Renmin Hospital of Wuhan University (n = 79). Healthy control (HC) participants (n = 78) of comparable age were recruited from local schools and hospitals. All participants were native Chinese speakers with sufficient comprehension and communication ability to complete the study.

Inclusion criteria for patients were as follows: (1) meeting the diagnostic criteria for depression disorder (DD) according to the Diagnostic and Statistical Manual of Mental Disorders, Fifth Edition (DSM-5) and confirmed using the Structured Clinical Interview for DSM-5 Research Version (SCID-5-RV); (2) age between 10 and 17 years; (3) first depressive episode; and (4) right-handedness. Healthy controls were screened by licensed psychiatrists through a brief face-to-face clinical interview to confirm the absence of clinically significant depressive or anxiety symptoms. Exclusion criteria for all participants were as follow: (1) severe or unstable medical or neurological conditions; (2) a history of schizophrenia or other major psychiatric disorders; and (3) contraindications to fNIRS recording. The study protocol was approved by the Ethics Committee of Renmin Hospital of Wuhan University (approval number: WDRY2024-K039). The study was an observational study without experimental intervention and registered at the Chinese Clinical Trial Registry (ChiCTR2400081102; URL: https://www.chictr.org.cn/showproj.html?proj=2400081102). Written informed consent was obtained from all participants and their legal guardians prior to study participation, in accordance with the Declaration of Helsinki.

### 2.2 Clinical and cognitive assessments

#### 2.2.1 Clinical assessments

Depression and anxiety symptoms were assessed using the 17-item Hamilton Depression Rating Scale (HAMD-17) and the Hamilton Anxiety Rating Scale (HAMA), respectively. Both instruments were administered by trained doctoral-level clinicians to ensure standardized evaluation and inter-rater reliability. Medication status, including current antidepressant use, was recorded at the time of fNIRS acquisition.

#### 2.2.2 Cognitive assessment (BACS)

Cognitive function was assessed using the Brief Assessment of Cognition in Schizophrenia (BACS), which evaluates verbal memory, working memory, motor speed, verbal fluency, attention and processing speed, and executive functioning. Although originally developed for schizophrenia, the BACS has been applied in mood disorders and healthy control samples, demonstrating sensitivity to cognitive differences in major depressive and bipolar disorders as well as normative populations (24–27). Raw BACS scores were used for group comparisons. For correlation analyses, raw scores were converted to standardized Z-scores relative to the mean and standard deviation of the healthy control group.

### 2.3 fNIRS data acquisition and processing

#### 2.3.1 Instrumentation and parameters

A 53-channel continuous-wave fNIRS system (BS-7000; Wuhan Znion Technology Co., Ltd., Wuhan, China) was used. The device consisted of 16 light emitters and 16 detectors, operating at two wavelengths (760 nm and 850 nm). The inter-optode distance was 3.0 cm, and the **s**ampling frequency was 20 Hz. The area between each emitter–detector pair defined a measurement channel, resulting in 53 channels in total. Optodes were positioned over the prefrontal scalp according to the international 10–20 system, covering the bilateral dorsolateral prefrontal cortex (DLPFC), Broca’s area, and the frontopolar area (FPA).

#### 2.3.2 Channel localization and ROI definition

Channels were grouped into predefined regions of interest (ROIs) based on their anatomical correspondence. To normalize channel locations, a three-dimensional digitizer (NirMap; Wuhan Znion Technology Co., Ltd., Wuhan, China) was used to record the spatial coordinates of four anatomical reference points (Nz, Cz, AL, RL) and 32 optodes (16 emitters and 16 detectors). Channel positions were transformed into Montreal Neurological Institute (MNI) space using NIRS-SPM and projected onto the cortical surface according to Brodmann areas. Based on maximum probability overlap, the 53 channels were assigned to six predefined ROIs, corresponding to the bilateral DLPFC, Broca, and FPA. The detailed channel-to-ROI mapping is provided in **Table 1**. ROI assignment was based on anatomical correspondence and probabilistic mapping, rather than single-channel inference.

**Table 1:** ROI definitions and corresponding fNIRS channels.

ROI	Left hemisphere channels	Right hemisphere channels
DLPFC	4, 6, 9, 10, 11, 12, 14, 15, 17, 18, 20, 24	26, 31, 32, 34, 38, 39, 40, 42, 43, 45, 47, 48
FPA	16, 19, 21, 22, 23, 27	30, 33, 35, 36, 37, 41
Broca	1, 2, 3, 5, 7, 8, 13	44, 46, 49, 50, 51, 52, 53

ROIs were defined based on maximum probability overlap with Brodmann areas. DLPFC, dorsolateral prefrontal cortex; FPA, frontopolar area.

#### 2.3.3 Resting-state procedure

Participants were seated upright in a comfortable chair in a quiet, dimly lit room. They were instructed to keep their eyes closed, remain awake and relaxed, and minimize head and body movement during the 6-min resting-state fNIRS recording.

#### 2.3.4 fNIRS data preprocessing

For resting-state fNIRS data, preprocessing was performed by Homer2 (28) and NIRS-KIT (29), two MATLAB-based toolboxes widely used for fNIRS analysis. The standardized preprocessing pipeline included the following steps: (1) Channel quality check. Bad channels were identified by calculating the coefficient of variation (CV) for each channel (30), channels with CV ≥ 25% was marked as invalid. If more than 20% of channels (≥ 12 of 53) were identified as bad for a subject, the dataset was excluded from further analysis. (2) Conversion to optical density. Raw light intensity signals were converted to optical density to linearize the Beer–Lambert relationship. (3) Motion artifact correction. Motion artifacts were identified and corrected using a cubic spline interpolation method (hmrMotionArtifactByChannel) with the follwing parameters: tMotion = 0.5 s, tMask = 1 s, STDEVthresh = 30, AMPthresh = 0.5 (31). (4) Temporal filtering. A band-pass filter (0.01–0.10 Hz) was applied to remove baseline drift, cardiac pulsations, and other physiological noise (32). (5) Hemoglobin concentration calculation. Optical density signals were converted to relative concentration changes of oxygenated hemoglobin (HbO), deoxygenated hemoglobin (HbR), and total hemoglobin using the modified Beer–Lambert law (33).Consistent with prior literature, HbO signals were selected as the primary indicator for subsequent analyses because of their higher signal-to-noise ratio and greater reliability for brain network metrics compared with HbR (19, 34, 35).

### 2.4 Resting state functional connectivity and fALFF analysis

We analyzed and compared resting-state functional connectivity (RSFC) between adolescent with depressive disorder (DD) and healthy controls (HC) at multiple levels, including channel-level spontaneous activity, channel- and ROI-level functional connectivity, global network topology, and their associations with clinical and cognitive measures.

#### 2.4.1 fALFF analysis

To characterize spontaneous regional brain activity, the fractional amplitude of low-frequency fluctuations (fALFF) was calculated at the channel level based on the preprocessed oxygenated hemoglobin (HbO) signals. Low-frequency oscillations within 0.01–0.08 Hz range are thought to reflect spontaneous neuronal activity (36). fALFF was computed as the ratio of the root mean square (RMS) of the power spectrum within the 0.01–0.08 Hz band to that across the whole spectrum (0.01–0.25 Hz), following previously established methods (37). Group differences in channel-level fALFF were examined using independent-samples t-tests. Channel-level fALFF findings were statistically analyzed at the level of individual channels, while their anatomical interpretation was informed by the predefined regions of interest (ROIs), to avoid overinterpretation of single-channel effects.

#### 2.4.2 Functional connectivity matrices

To visualize the whole whole-network connectivity patterns, we constructed correlation matrices by computing the Pearson correlation coefficient (r) between all possible channel pairs. Fisher’s r-to-z transformation was used to normalize correlation coefficients prior to subsequent group-level statistical analyses, ensuring compliance with parametric statistical assumptions.

#### 2.4.3 ROI-based connectivity and edge number analysis

To obtain region-level functional connectivity, the original 53×53 channel-wise functional connectivity (FC) matrices were aggregated into 6×6 ROI-based matrices by averaging Fisher z-transformed correlation coefficients across all channel pairs belonging to each ROI pair (left/right DLPFC, FPA, Broca’s area). Based on these ROI matrices, functional connections were categorized following Zhu et al. (19) into four types: (i) SC-I (short-distance I): intrahemispheric within the same ROI (e.g., within DLPFC-L, FPA-R, or Broca-L); (ii) SC-II (short-distance II): intrahemispheric between different ROIs (e.g., DLPFC-L–FPA-L, FPA-R–Broca-R); (iii) LC-I (long-distance I): interhemispheric homologous connectivity (e.g., DLPFC-L–DLPFC-R, FPA-L–FPA-R); (iv) LC-II (long-distance II): interhemispheric heterologous connectivity (e.g., DLPFC-L–FPA-R, Broca-L–DLPFC-R). For each subject, ROI-to-ROI Pearson correlation coefficients were calculated and transformed to z-values using Fisher’s r-to-z transformation. To ensure robust estimation of network properties, correlation matrices were binarized across a range of thresholds (T = 0.40–0.90, step size = 0.05), with edges defined according to threshold-specific inclusion criteria applied to the Fisher z-transformed connectivity values. At each threshold, the number of edges retained in each connectivity category (SC-I, SC-II, LC-I, LC-II) was then counted for each participant.

#### 2.4.4 Graph-theoretical global metrics

To capture topological properties of brain networks, we used the GRETNA toolbox (38; MATLAB, Mathworks, Natick, MA, USA) to compute global network metrics, including normalized clustering coefficient (Cp), characteristic path length (Lp), global efficiency (Eglob), local efficiency (Eloc). These metrics provide indices of network segregation and integration, consistent with prior graph-theory studies (6, 39). As in the ROI-based analysis, functional connectivity matrices were thresholded across a range of correlation values (T = 0.40 – 0.90, step = 0.05), and metrics were computed at each threshold. This threshold-dependent approach allowed evaluation whether group differences in global topology were consistent across multiple network densities. Group effects on these metrics across thresholds were subsequently assessed using mixed-model analyses.

### 2.5 Statistical analysis

**Figure 1:**
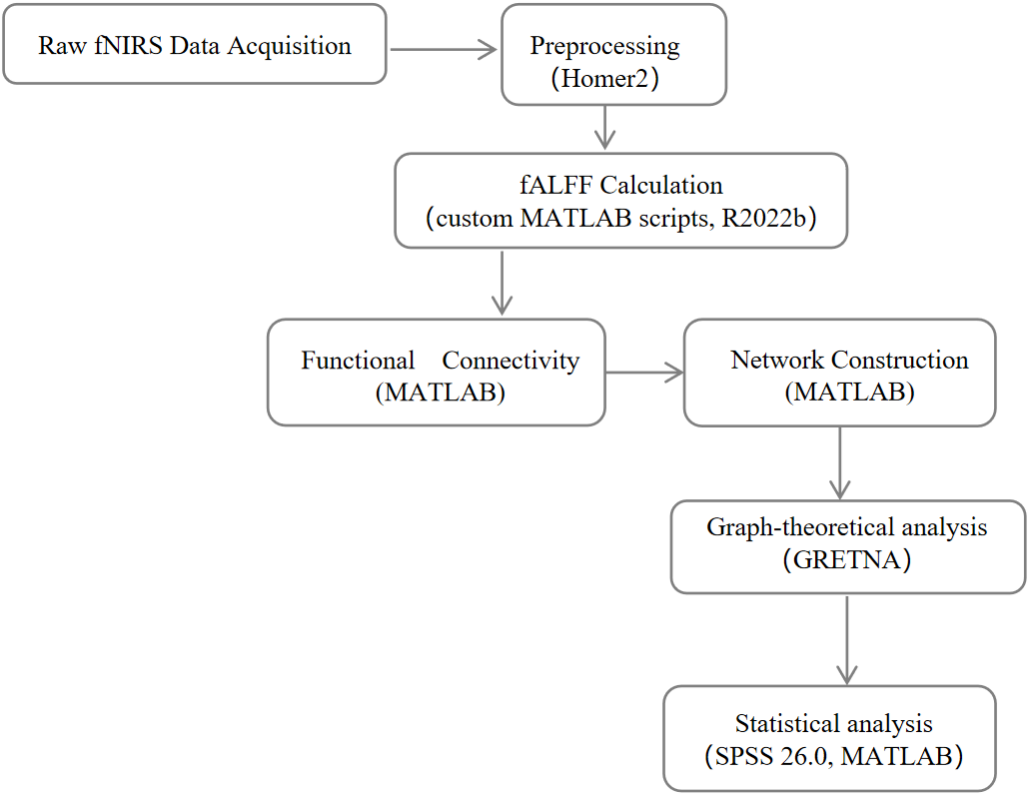
Analysis pipeline for resting-state fNIRS data processing and network analysis.

All analyses were conducted in SPSS 26.0 (IBM Corp., Armonk, NY, USA) and MATLAB R2022b (MathWorks, Natick, MA, USA) as shown in **Figure 1**.

#### 2.5.1 Group comparisons

Within-group consistency of functional connectivity (FC) was assessed using one-sample t-tests, and between-group differences in fALFF, ROI connectivity, and global network metrics were examined using independent-samples t-tests.

#### 2.5.2 Threshold-based network analysis

Group differences in edge counts across thresholds were tested using mixed-model ANOVA, with group (DD vs. HC) as the between-subject factor, threshold (11 levels, T = 0.40–0.90, step = 0.05) as the repeated-measures factor, and connectivity type (SC-I, SC-II, LC-I, LC-II) as the within-subject factor. Similarly, group effects on global graph-theoretical metrics across thresholds were assessed using mixed-effect models, consistent with standard network analysis procedures.

#### 2.5.3 Brain–behavior correlation analyses

Pearson or Spearman correlations analyses were conducted to examine associations between fNIRS indices (fALFF, ROI connectivity, and global network metrics) and cognitive performance (BACS subtests), depending on data distribution. In addition, partial correlations controlling for depressive (HAMD-17) and anxiety (HAMA) severity were performed to determine whether observed associations were independent of symptom burden. All correlations were interpreted with reference to channel-level fALFF, anatomical ROIs, or global network topology, as appropriate, to avoid overinterpretation based on individual channels.

Multiple comparisons for fNIRS-derived metrics were controlled using the Benjamini–Hochberg false discovery rate (FDR) procedure. All reported p-values for these analyses are FDR-corrected. Statistical significance was set at two-tailed p < 0.05.

## 3 Results

### 3.1 Demographic characteristics of participants

As shown in **Table 2**, a total of 157 participants aged 10–17 years were included in the present study. No significant differences were observed between the depressive disorder (DD) and healthy control (HC) groups in sex, age, height, weight, or body mass index (BMI) (all p > 0.05). In contrast, participants in the DD group exhibited significantly higher sores on the HAMD-17 and HAMA compared with HCs (all p < 0.001).

**Table 2:** Demographics and clinical measures (DD vs HC).

Variables	DD group (n = 79)	HC group (n = 78)	P-value
Sex (male/female)	28/51	24/54	0.651
Age (years)	14.81 ± 1.61	14.17 ± 1.74	0.791
Education (years)	9.03 ± 1.71	8.9 ± 1.6	0.087
Height (cm)	164.85 ± 7.06	164.15 ± 8.45	0.602
Weight (kg)	55.89 ± 12.98	57.72 ± 10.75	0.372
BMI (kg/m²)	20.47 ± 3.99	21.40 ± 3.74	0.160
HAMD	19.5 (14.25, 25)	1 (0, 3)	< 0.001
HAMA	17 (13, 21)	1 (1, 4)	< 0.001

Values are presented as mean ± SD or median (Q1–Q3) depending on distribution. Group differences were tested with independent-samples t-tests (for normally distributed variables), Mann–Whitney U tests (for non-normally distributed variables), or χ² tests (for categorical variables).

Within the DD group, the mean illness duration was approximately 21 months. At the time of assessment, 34 patients (43.03%) were receiving antidepressant treatment—most commonly selective serotonin reuptake inhibitors (SSRIs)—and 31 (39.24%) were first-episode cases (see **Table 3** for details).

**Table 3:** Clinical characteristics of adolescents with depression variable.

Variable	DD group (n = 79)
Illness duration (months), mean ± SD	20.57 ± 14.78
First-episode cases, n (%)	31 (39.24%)
Recurrent cases, n (%)	48 (60.76%)
Medication status	
Receiving antidepressants, n (%)	34 (43.03%)
Medication-naïve, n (%)	45 (56.97%)
Medication type†	
Sertraline, n	13
Fluvoxamine, n	6
Fluoxetine, n	5
Others‡, n	10

DD, depressive disorder; Medication status was assessed at the time of fNIRS scanning.

^†^Medication status was assessed at the time of fNIRS scanning.

^‡^Others include [e.g., escitalopram / venlafaxine / duloxetine] (if applicable).

As shown in **Table 4**, DD patients demonstrated significantly poorer performance than HCs across all BACS cognitive domains, including verbal memory, working memory, motor speed, verbal fluency, attention and processing speed, and executive functioning (all p < 0.05).

**Table 4:** Group differences in cognitive performance assessed by the brief assessment of cognition in Schizophrenia (BACS).

BACS Domain	DD group (mean ± SD)	HC group (mean ± SD)	T value	P value
Verbal Memory	42.48 ± 10.40	45.04 ± 9.70	-1.60	< 0.001
Working Memory	21.52 ± 3.75	23.70 ± 2.85	-4.11	0.018
Motor Speed	65.69 ± 12.50	70.40 ± 11.60	-2.45	< 0.001
Verbal Fluency	27.81 ± 9.30	31.1 ± 8.42	-2.32	< 0.001
Attention and Processing Speed	56.00 ± 13.56	60.03 ± 11.3	-2.03	< 0.001
Executive Functioning	17.20 ± 2.55	18.7 ± 1.91	-4.18	0.020

BACS, Brief Assessment of Cognitionin Schizophreia. Raw scores are reported; Z-scores (standardized to the HC group) were used for correlation analyses.

### 3.2 fALFF

**Figure 2:**
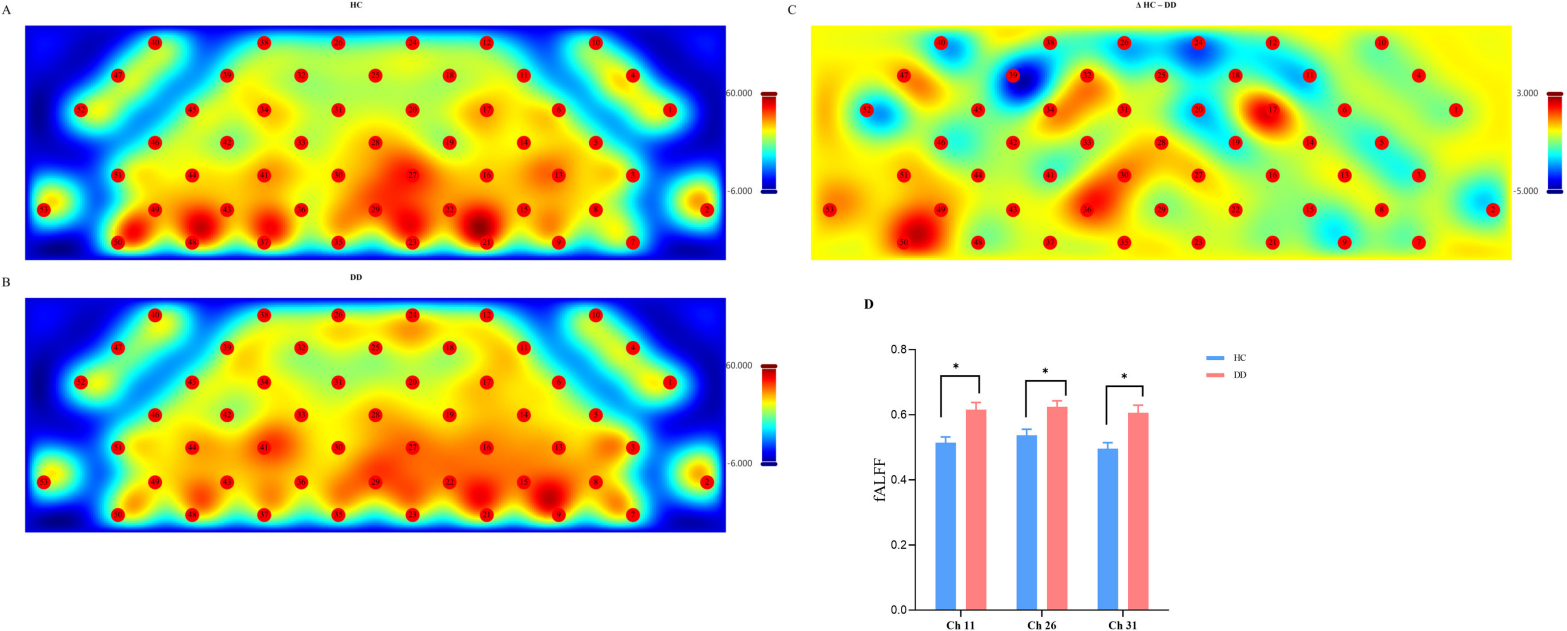
fALFF analysis. Distribution of fALFF values across 53 channels in HC group **(A)**, in DD group **(B)**; the color bar indicates fALFF values **(C)**. **(D)** Group comparisons of fALFF values in the three channels showing significant differences (ch11, ch26, ch31; mean ± SE). The asterisk indicates a significant difference after FDR correction (^*^p < 0.05).

Group comparisons of spontaneous brain activity revealed elevated Falff in the DD group relative to the HC group. As shown in **Figure 2**, average fALFF values across channels was significantly higher in DD group. Channel-wise analyses further indicated significantly increased fALFF in DD at ch11 (t = 3.63, p _FDR_= 0.013), ch26 (t = 3.37, p _FDR_ = 0.020), and ch31 (t = 3.82, p _FDR_= 0.013).

### 3.3 Correlation matrixes

**Figure 3:**
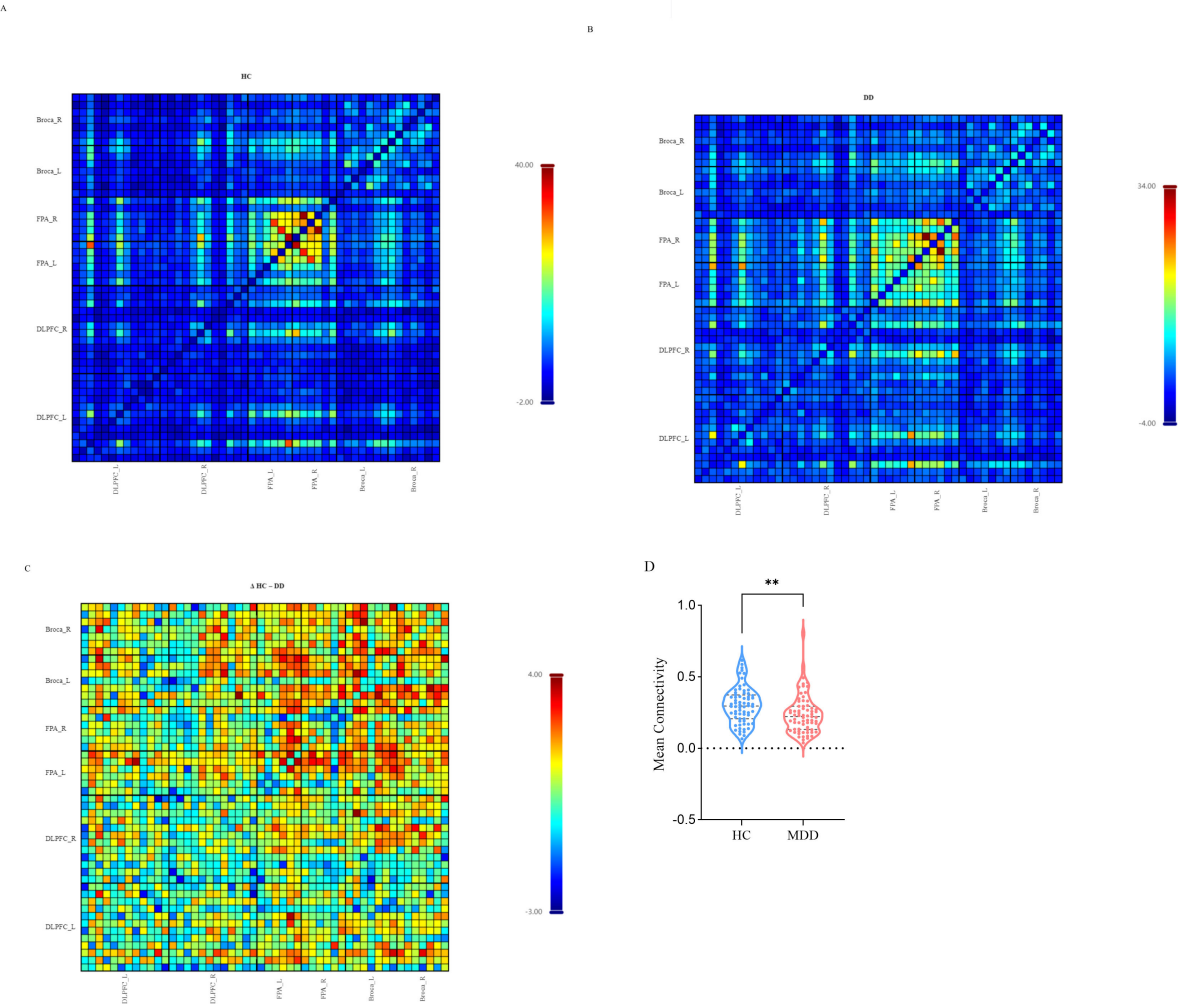
Group-level functional connectivity (FC) correlation matrices of the prefrontal cortex. **(A)** Group-averaged FC matrix in the HC group. **(B)** Group-averaged FC matrix in the DD group. **(C)** Difference matrix (Δ HC – DD) showing group-level contrasts. Each pixel represents the Pearson correlation coefficient (r) of the corresponding channel pair within or between ROIs. L, left hemisphere; R, right hemisphere; DLPFC, dorsolateral prefrontal cortex, FPA, frontopolar area. **(D)** Group-averaged mean FC strength in HC and DD. ** indicates P < 0.01.

Group-level HbO correlation matrices are shown in **Figures 3A–C** for the HC group, DD group, and the group difference (ΔHC–DD). Compared with the HC group, the DD group exhibited markedly reduced resting-state functional connectivity (RSFC) across prefrontal regions. As shown in **Figure 3D**, mean FC strength was significantly lower in the DD group (M ± SD, 0.24 ± 0.02) than in the HC group (0.30 ± 0.02) (t = –2.75, p = 0.007).

### 3.4 Four clusters of functional connectivities

**Figure 4:**
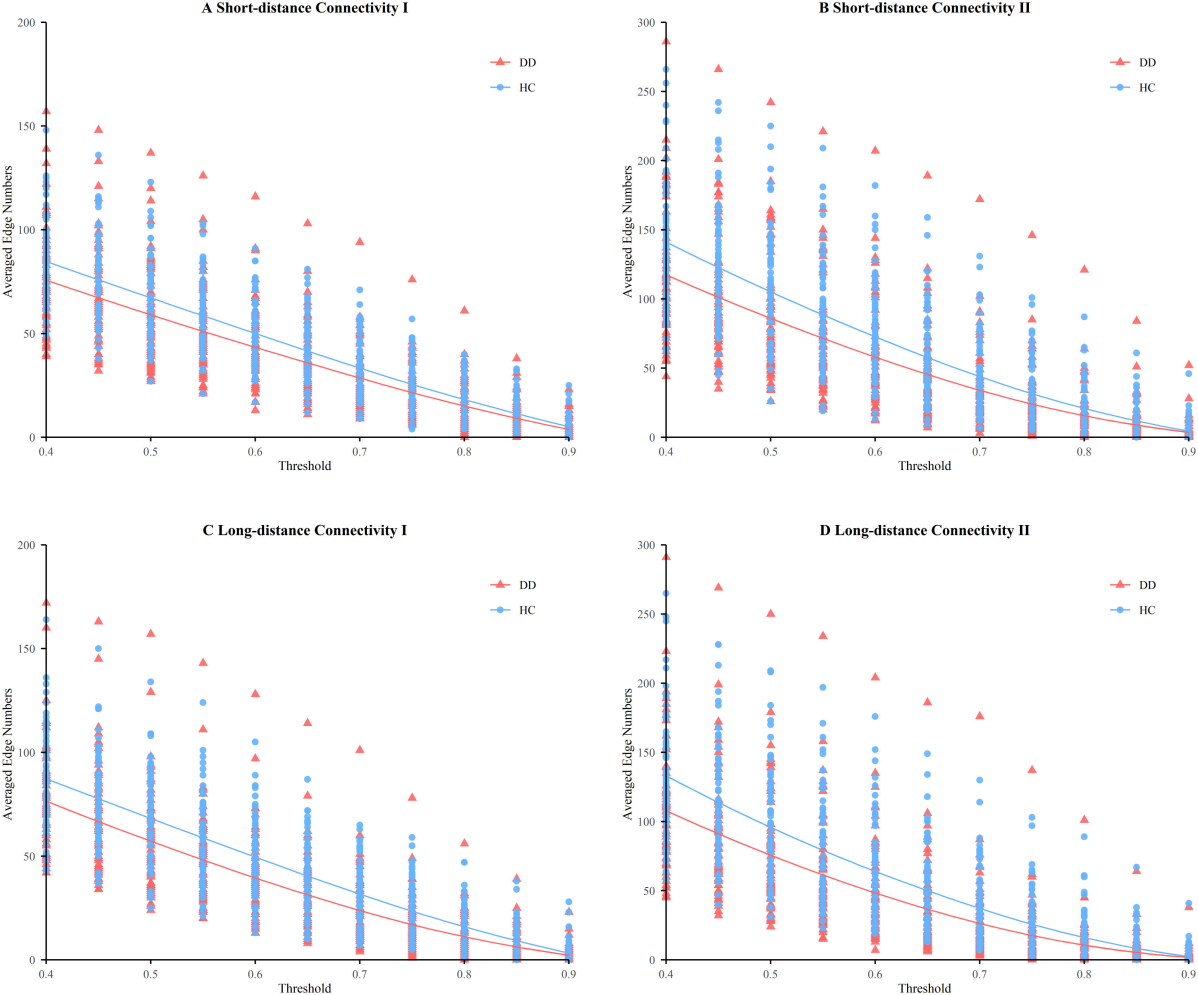
Four clusters of functional connectivities among HC and DD: **(A)** Short-distance Connectivity I; **(B)** Short-distance Connectivity II; **(C)** Long-distance Connectivity I; **(D)** Long-distance Connectivity II. Each data point represents the averaged edge number for each participant at a certain threshold value.

**Figure 4** shows the results of the four clusters of functional connectivity clusters in the HC and DD groups. Each data point represents the averaged edge number for each participant at a given threshold, and the curves illustrate changes in connection numbers across thresholds. As the threshold increased, the number of edges decreased.

**Table 5** summarizes the mixed model ANOVA results. Significant main effects of both Group and Threshold were observed across SC-I, SC-II, LC-I, and LC-II. In all four clusters, the DD group exhibited significantly reduced connectivity relative to the HC group, with robust group effects in SC-I (F (1, 1485) = 43.84, p _FDR_ < 0.001), SC-II (F (1, 1485) = 54.33, p _FDR_ < 0.001), LC-I (F (1, 1485) = 71.35, p _FDR_ < 0.001), and LC-II (F (1, 1485) = 63.45, p _FDR_ < 0.001). Significant main effects of Threshold were observed for all clusters (all p _FDR_ < 0.001). .

**Table 5:** Mixed-model ANOVA results for edge numbers across connectivity clusters (SC-I, SC-II, LC-I, LC-II).

Cluster	Comparison	F-value	Num. DF	Denom. DF	p _FDR_
SC-I	Group	43.84	1	1485	< 0.001
	Threshold	332.25	10	1485	< 0.001
	Group × Threshold	0.92	10	1485	0.517
SC-II	Group	54.33	1	1485	< 0.001
	Threshold	234.49	10	1485	< 0.001
	Group × Threshold	1.90	10	1485	0.041
LC-I	Group	71.35	1	1485	< 0.001
	Threshold	308.01	10	1485	< 0.001
	Group × Threshold	1.34	10	1485	0.203
LC-II	Group	63.45	1	1485	< 0.001
	Threshold	222.42	10	1485	< 0.001
	Group × Threshold	2.28	10	1485	0.012

F-Value, The test statistic value corresponding to the L matrix used for testing the model term; Num. DF, The numerator degrees of freedom for the F test. Denom; DF, The denominator degrees of freedom for the F test; *p* values were FDR-corrected. *p < 0.05, **p < 0.01; ***p < 0.001.

Main effects of Threshold were also observed for all clusters (all p _FDR_ < 0.001), reflecting expected variations in connectivity strength. Importantly, significant Group × Threshold interactions were detected for SC-II (F (10, 1485) = 1.90, p _FDR_ = 0.041) and LC-II (F (10, 1485) = 2.28, p _FDR_ = 0.012), suggesting that group differences in short- and long-distance connectivity were partly threshold-dependent. By contrast, no significant interactions were found for SC-I (p _FDR_ = 0.517) or LC-I (p _FDR_ = 0.203).

**Figure 5:**
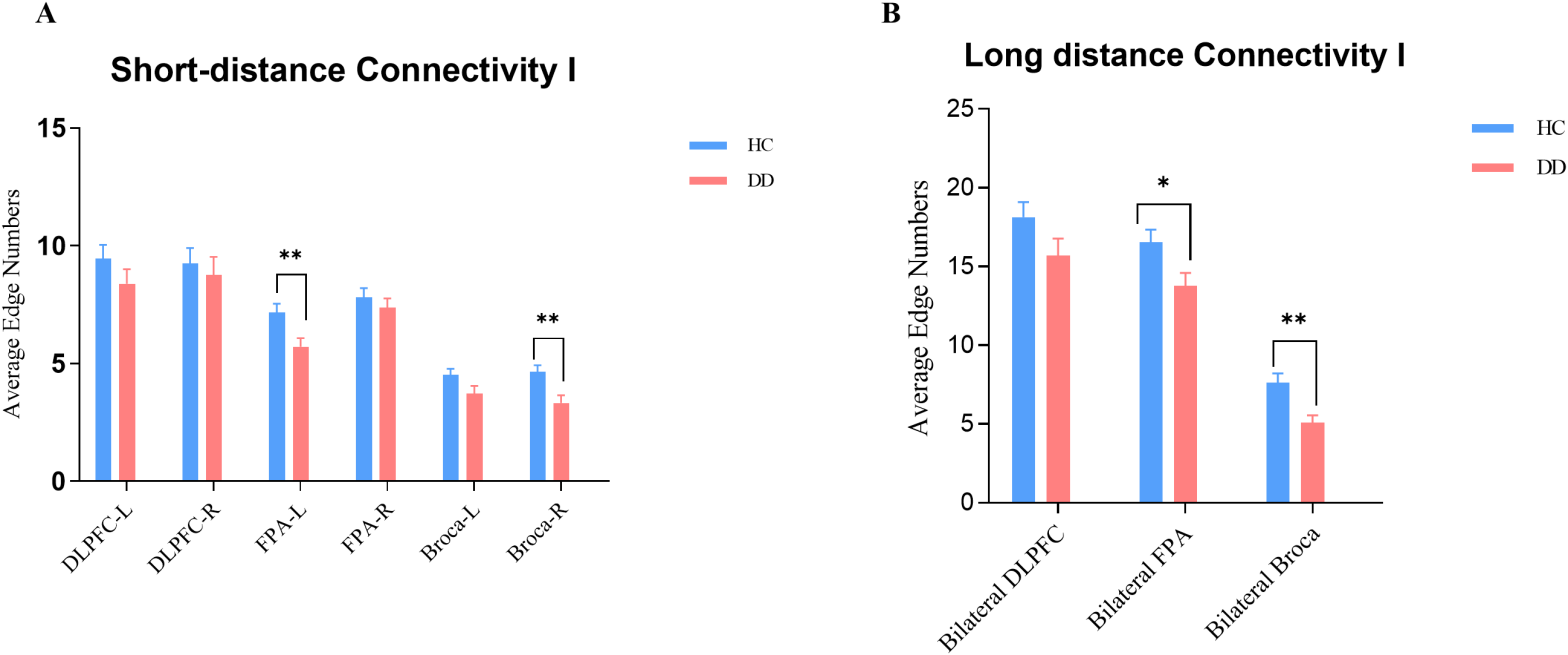
ROI-based connectivity differences in SC-I and LC-I. **(A)** Short-distance connectivity I (SC-I) within individual ROIs. **(B)** Long-distance connectivity I (LC-I) between bilateral DLPFC, FPA, and Broca. Bars represent group-averaged edge numbers (mean ± SE). Asterisks denote significant group differences (*p < 0.05; **p < 0.01).

To further examine group differences in SC-I and LC-I, connectivity patterns were examined at by the ROIs level (**Figure 5**). For SC-I, significant reductions were observed in the FPA-L and Broca-R, with intra-regional connectivity in these ROIs was significantly lower in the DD group compared with the HC group; no significant differences were found in the other ROIs (**Figure 5A**). For LC-I, significant reductions were detected in the bilateral FPA and bilateral Broca, with interhemispheric symmetrical connectivity markedly lower in the DD group than in the HC group, whereas no significant difference was observed in the bilateral DLPFC (**Figure 5B**).

### 3.5 Global network metrics

**Figure 6:**
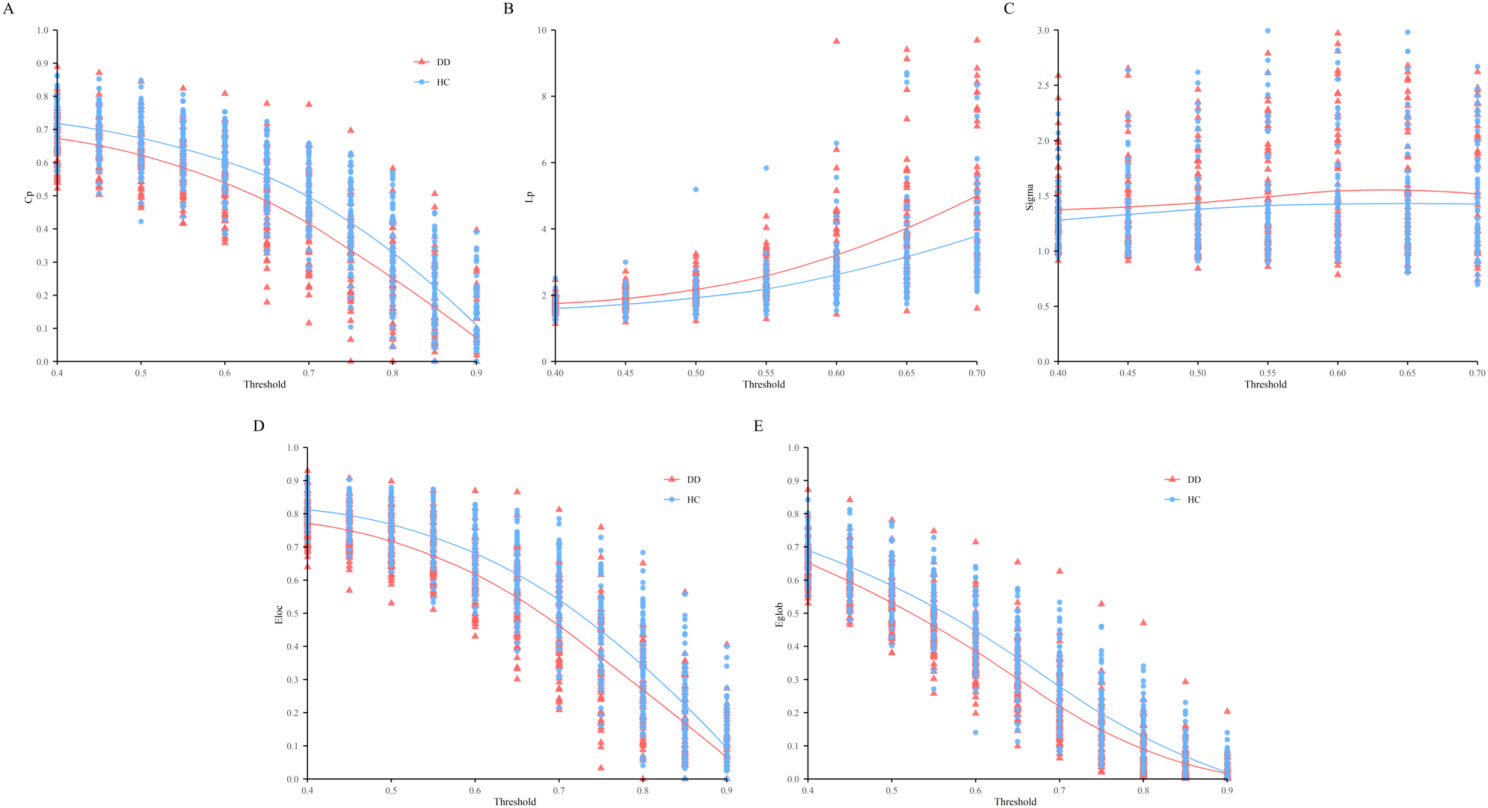
The global network metrices in a range of T values (0.4-0.9). **(A)** C_P_, the clustering coefficient; **(B)** L_P_, the characteristic path length, **(C)** Simga, **(D)** the local efficiency, **(E)** the global efficiency. The shadows indicate standard error in all participants.

To further characterize the intrinsic organization of cortical networks, we examined global network properties in both groups, focusing on the clustering coefficient (Cp), characteristic path length (Lp), and global and local efficiency (Eglob, Eloc), as a range of thresholds (T = 0.40 – 0.90, step = 0.05). As shown in **Figure 6**, compared with the HC group, the DD group exhibited lower Cp, reduced Eloc and Eglob, and higher Lp. The small-worldness index (sigma) did not differ significantly between groups. As shown in **Table 6**.

**Table 6:** Mixed-model ANOVA results for global network metrics across thresholds (Cp, Lp, simga local/global efficiency).

Response variable	Comparison	F-value	Num. DF	Denom. DF	p FDR
Gamma	Group	4.46	1	1485	0.035
	Threshold	17.94	10	1485	< 0.001
	Group × Threshold	0.47	10	1485	0.91
Lambda	Group	2.44	1	1485	0.119
	Threshold	68.32	10	1485	< 0.001
	Group × Threshold	1.90	10	1485	0.005
Simga	Group	2.73	1	1485	0.99
	Threshold	12.25	10	1485	< 0.001
	Group × Threshold	0.47	10	1485	0.91
Local efficiency	Group	127.86	1	1485	< 0.001
	Threshold	802.66	10	1485	< 0.001
	Group × Threshold	1.18	10	1485	0.299
Global efficiency	Group	117.55	1	1485	< 0.001
	Threshold	1182.41	10	1485	< 0.001
	Group × Threshold	1.96	10	1485	0.04

*p < 0.05; **p < 0.01; ***p < 0.001.

Mixed-model ANOVA revealed a significant main effect of Group on Cp (F (1, 1485) = 4.46, p _FDR_ = 0.035), indicating lower Cp in the DD group relative to the HC group, while no significant Group × Threshold interaction emerged (p _FDR_ = 0.91). For Lp, no significant main effect of Group was detected (p _FDR_ = 0.119); however, a significant Group × Threshold interaction was observed (F (10, 1485) = 1.90, p _FDR_ = 0.005), indicating threshold-dependent group differences. For small-worldness (sigma), neither the main effect of Group (p _FDR_ = 0.99) nor the Group × Threshold interaction (p _FDR_ = 0.91) was observed, indicating no significant group differences in small-worldness across thresholds.

Both local efficiency (F (1, 1485) = 127.86, p _FDR_ < 0.001) and global efficiency (F (1, 1485) = 117.55, p _FDR_ < 0.001) were significantly lower in the DD group compared with the HC group. Moreover, global efficiency showed a modest but significant Group × Threshold interaction (F (10, 1485) = 1.96, p _FDR_ = 0.040), indicating that group differences in integrative efficiency varied across network sparsity levels.

### 3.6 Correlational analysis of resting state functional connectivity, BACS and clinical symptoms

We found that the fALFF in channel 11 (ch11) was positively correlated with verbal fluency performance on the BACS (r = 0.54, p < 0.001). This association remained significant after controlling for depressive (HAMD-17) and anxiety (HAMA) symptom severity (partial r = 0.48, p = 0.006). In contrast, the fALFF in channel 31 (ch31) showed a negative correlation with executive function (r = - 0.34, p = 0.044), which also remained significant after adjusting for HAMD-17 and HAMA symptom severity (partial r = - 0.37, p = 0.042). Importantly, none of the fALFF or global network metrics that differed between groups were significantly correlated with depression severity (HAMD-17) or anxiety (HAMA) symptom severity.

## 4 Discussion

In the present work, we used fNIRS to investigate both regional spontaneous activity and global network properties of the prefrontal cortex during resting state in adolescents with depression compared with healthy controls. Amplitude analyses revealed significantly increased fALFF values in multiple prefrontal channels (e.g., ch11, ch26, ch31) in patients with depression, indicating altered intrinsic spontaneous neural activity. Resting-state functional connectivity demonstrated reduced average connectivity strength, particularly within the prefrontal cortex and between bilateral frontal regions, including the frontal pole area (FPA) and Broca’s area. At the network level, graph-theoretical analyses showed significantly lower clustering coefficient (Cp), local efficiency (E_loc), and global efficiency (E_glob) in patients with depression, whereas characteristic path length (Lp) and small-worldness (sigma) did not differ between groups, suggesting impaired functional segregation and integration despite preservation of the overall small-world architecture. Moreover, a significant group × threshold interaction was observed, suggesting that group differences in network topology became more pronounced under higher sparsity conditions. Finally, brain–behavior correlation analyses showed that fALFF in ch11 was positively associated with verbal fluency performance on the BACS, while fALFF in ch26 was negatively correlated with Tower of London performance; importantly, these associations remained significant after controlling for depressive (HAMD-17) and anxiety (HAMA) symptom severity, indicating that altered spontaneous prefrontal activity was associated with cognitive performance independently of depressive and anxiety symptom severity. Taken together, these findings suggest selective, network-level alterations within the prefrontal cortex in adolescents with depression, rather than a global breakdown of functional brain organization.

Our findings converge with and extend previous work on resting-state abnormalities in depression. In adolescent and first-episode samples, prior fMRI studies have consistently reported reduced inter-network and interhemispheric connectivity, as well as evidence of disrupted small-world organization and decreased efficiency. For example, attenuated fronto-limbic and default mode connectivity, as well as global network inefficiency, have been reported in adolescent samples with depression, whereas altered small-world network organization has been demonstrated in first-episode, drug-naïve patients (22, 40, 41). In adult and mixed-age samples, although findings have been more heterogeneous, several studies have documented lower clustering coefficient (Cp) and local efficiency (E_loc), alongside alterations in global efficiency (E_glob) and characteristic path length (Lp) (42, 43). Taken together, these convergent findings across age groups suggest that reduced functional segregation and impaired network integration represent robust features of depression, while the degree of small-world alteration may vary with sample characteristics and illness stage. At the regional level, resting-state fMRI studies have consistently reported altered fALFF in depression, including increases in prefrontal and insular regions and associations with executive dysfunction or symptom severity (13, 44, 45). Consistent with these findings, we observed altered prefrontal fALFF in adolescents with depression, with channel-level fALFF showing significant associations with cognitive performance.

Importantly, prior fNIRS studies provide convergent evidence for prefrontal network abnormalities in depression. Resting-state work has reported reduced prefrontal connectivity and disrupted network topology in affective disorders (19), while task-based studies in adolescents with depression have demonstrated attenuated prefrontal activation and functional connectivity during cognitive tasks (20, 46). Consistently, decreased connectivity within the cognitive control network has also been observed in MDD (47).

From a network perspective, our graph-theoretical findings—lower Cp, E_ loc, E_ glob, with preserved small-worldness—suggest impaired functional segregation and integration in adolescents with depression. Specifically, local information processing and network robustness appear reduced, while large-scale information transfer is less efficient, despite preservation of the overall small-world architecture. This interpretation is consistent with the canonical mapping of graph metrics to segregation (Cp, E_ loc) and integration (Lp, E_ glob) in brain networks (48), and align with meta-analytic evidence of large-scale network inefficiency in MDD (10). At the regional level, we additionally observed increased fALFF in prefrontal region, reflecting altered low-frequency spontaneous activity. fALFF quantifies the fraction of low-frequency power relative to the whole spectrum and is widely used to index intrinsic activity of cortical regions at rest (12). Previous work has shown that prefrontal fALFF alterations are associated with cognitive dysfunction in first-episode, drug-naïve patients with MDD (13) and to symptom severity in clinical samples (44), providing context for the behavioral relevance of our fALFF findings in adolescents.

Bridging regional and network levels, network efficiency has been robustly associated with general cognitive capacity in humans (14). Convergent evidence across modalities helps contextualize the present findings. At rest, prior fNIRS studies have reported reduced prefrontal functional connectivity and disrupted network topology in affective disorders (19), consistent with the prefrontal inefficiency observed here. In our adolescent cohort, this network-level inefficiency co-occurred with elevated prefrontal fALFF, suggesting altered spontaneous low-frequency activity at the regional level. Importantly, these alterations showed region-specific associations with cognition: fALFF in channel 11 (left DLPFC) was positively correlated with verbal fluency, whereas fALFF in channel 31 (DLPFC-R) was negatively correlated with Tower of London performance. Together, these findings indicate that increased regional spontaneous activity may relate differentially to cognitive performance across prefrontal subregions, while reduced network efficiency reflects a broader vulnerability in large-scale functional organization. Thus, the co-occurrence of altered regional activity and reduced network efficiency may reflect atypical integration of prefrontal systems in adolescents with depression.

Importantly, a significant group × threshold interaction was observed in global network metrics, indicating that the between-group differences varied as a function of network sparsity. As the threshold increased and the network became sparser, efficiency-related measures declined more steeply in the depression group, suggesting reduced robustness in maintaining efficient information integration. The threshold-dependent pattern suggests greater sensitivity of network efficiency to increasing sparsity in adolescents with depression, indicating reduced robustness in maintaining efficient information integration under constrained connectivity conditions. Such an interpretation is broadly consistent with prior fMRI and graph-theoretical studies reporting altered network resilience, efficiency, or flexibility in depressive disorders (6, 48). Taken together, the present results suggest that an imbalance between functional segregation and integration may contribute to large-scale organizational alterations associated with cognitive and emotional dysfunction in adolescent depression.

Adolescence represents a critical period for prefrontal and large-scale network maturation, characterized by prolonged synaptic pruning and myelination extend into early adulthood (49, 50). During typical development, brain networks progressively shift from locally biased toward more distributed configurations while preserving small-world organization (6). Structural and multimodal imaging studies further indicate that adolescence is characterized by increasing network modularity and strengthening structure–function coupling in transmodal regions, particularly the prefrontal cortex, supporting the maturation of executive functions (7, 8). In parallel, improvements in cognitive control, including working memory and inhibitory processes, track the protracted development of frontoparietal circuitry (51, 52). Against this normative developmental backdrop, our observation of reduced clustering and efficiency in adolescents with depression may reflect a deviation from typical network maturation, suggesting altered specialization and integration during a period critical for cognitive consolidation.

A strength of the present study is the integration of neurocognitive measures (BACS) with resting-state fNIRS metrics, which remains relatively underexplored in adolescent depression research. We found that prefrontal fALFF was associated with verbal fluency and Tower of London performance, indicating that spontaneous neural activity at rest is associated with individual differences in cognitive function. This finding is consistent with prior evidence showing that resting-state network properties are linked to individual differences in cognition. For example, global network efficiency has been associated with intellectual performance (14), and flexible connectivity patterns have been shown to support cognitive control (53). In addition, resting-state fMRI studies have demonstrated that fALFF in frontal and frontoparietal regions correlates with executive and working memory performance (54, 55). Together, these findings suggest that altered prefrontal fALFF in adolescent depression may have cognitive relevance, whereas reduced network efficiency may reflect a more global systems-level vulnerability.

From a translational perspective, these findings support the feasibility of fNIRS as a developmentally appropriate tool for probing intrinsic brain dynamics in adolescents with depression. However, further studies with larger samples and longitudinal designs are required before clinical relevance can be established.

## 5 Limitations and future directions

Several limitations of the present study should be noted. First, the sample size, though comparable to other adolescent neuroimaging studies, was modest and may limit generalizability; replication in larger, multi-site cohorts is needed. Second, the fNIRS montage covered only prefrontal regions, preventing inferences about distributed networks relevant to depression. Third, the cross-sectional design precludes causal conclusions regarding whether altered fALFF and reduced network efficiency reflect vulnerability, state effects, or sequelae; longitudinal studies are required. Finally, although resting-state indices were linked to BACS performance, task-based fNIRS paradigms and multimodal imaging (e.g., fMRI, EEG) will be important to confirm functional relevance and cross-method robustness. Future research should therefore longitudinal designs, developmental stratification, and predictive modeling approaches to establish the reliability and clinical utility of resting-state fNIRS markers in adolescent depression.

## 6 Conclusion

This study provides evidence that adolescents with depression exhibit elevated prefrontal fALFF and reduced network efficiency during the resting state as measured by fNIRS. Importantly, fALFF was significantly associated with BACS performance, linking spontaneous prefrontal activity to cognitive function in domains such as verbal fluency and executive function. By contrast, reduced network efficiency did not directly correlate with cognition, suggesting it may represent a broader systems-level alteration. Taken together, these findings indicate that resting-state fALFF is a developmentally sensitive marker of altered brain–cognition coupling, while reduced network efficiency reflects broader organizational disruptions. While methodological refinements and longitudinal validation are required, these results advance our understanding of the neurobiological underpinnings of adolescent depression and support the potential of fNIRS as a practical tool for probing early brain dysfunction in youth.

## Data Availability

The raw data supporting the conclusions of this article will be made available by the authors, without undue reservation.
