# Supplementary material for: Altered resting-state prefrontal activity and network topology in adolescents with depression: an fNIRS study
Source: Front Psychiatry. 2026 Feb 23;17:1696556. doi: 10.3389/fpsyt.2026.1696556 (PMC12968784; doi:10.3389/fpsyt.2026.1696556)
Supplement: Supplementary file 1 [file Table1.docx]

Supplementary

Method S1. fALFF calculation

fALFF values were calculated using custom-written MATLAB scripts. For each participant and each channel, the preprocessed HbO time series was linearly detrended. Power spectral density was estimated using an FFT-based method. The root mean square (RMS) of the power spectrum within the 0.01–0.08 Hz band was divided by the RMS of the full frequency band (0.01–0.25 Hz) to obtain channel-wise fALFF values.

The following MATLAB code illustrates the core computational steps of fALFF estimation; data loading and preprocessing steps are omitted for clarity.

% fALFF_calculation.m

% This script illustrates the core procedure used to calculate

% fractional amplitude of low-frequency fluctuations (fALFF)

% from preprocessed fNIRS time series data.

%

% Input:

% time_series: a vector containing the HbO signal of one channel

% fs: sampling rate (Hz)

%

% Output:

% fALFF value for the input time series

function fALFF = fALFF_calculation(time_series, fs)

% Remove linear trend

time_series = detrend(time_series);

% Compute power spectrum using FFT

N = length(time_series);

Y = fft(time_series);

P = abs(Y).^2 / N;

% Frequency vector

freq = (0:N-1) * fs / N;

% Define frequency bands

low_band = (freq >= 0.01) & (freq <= 0.08);

full_band = (freq >= 0.01) & (freq <= 0.25);

% Compute fALFF

fALFF = rms(P(low_band)) / rms(P(full_band));

end
